# Effect of selenium hyperaccumulation on the root endophytic and rhizosphere microbiome of two *Astragalus* species

**DOI:** 10.1111/plb.70263

**Published:** 2026-07-13

**Authors:** C. Dotson, C. Kurowski, M. Grillo

**Affiliations:** ^1^ Department of Biology Loyola University Chicago Chicago Illinois USA

**Keywords:** *Astragalus*, heavy metal hyperaccumulation, microbiome, plant–microbe, rhizobia, selenium

## Abstract

- Metal hyperaccumulation is prevalent throughout plant evolution, particularly in the legume family (Fabaceae), and acts as a presumed chemical defence against herbivory. However, metal hyperaccumulation can have non‐target impacts on other biological interactors, including plant–microbe interactions. This information is important given the interest in utilizing hyperaccumulating plants for phytoremediation of anthropogenically contaminated soils.
- Here we employ a greenhouse experiment manipulating selenium level along with 16S rRNA gene amplicon sequencing methods to explore the effect of selenium on the prokaryotic microbiome of the selenium hyperaccumulator *Astragalus crotalariae* and non‐accumulator *A. lentiginosus* var. *borreganus.*
- Regardless of hyperaccumulator status, both plant species accumulated high levels of selenium in leaf tissue when grown on soils with high selenium levels. The effect of selenium on the prokaryotic communities was slightly more pronounced in *A. lentiginosus* than in *A. crotalariae*, explaining ~5% more of the observed variation. This effect of selenium addition is seen most prevalently in *A. lentiginosus* root endosphere communities, in which selenate treatment impacted alpha diversity and whole community composition. Many individual microbes were affected by selenium addition; notably, an ASV identified as *Allomesorhizobium*, the nodulation‐inducing genera of *Astragalus* spp., appeared in significantly less abundance in roots of *A. lentiginosus* plants grown on highly seleniferous soils.
- This project highlights the potential significance of ecological partners in metal accumulating plants and the necessity of their consideration when using these plants for phytoremediation.

## Graphical abstract

Selenium hyperaccumulation has little impact on the overall microbiome diversity but rather affects interactions with key plant microbial mutualists, including rhizobia.

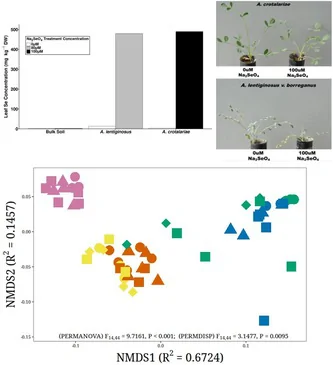

## INTRODUCTION

Plants display an incredible diversity of chemical compounds, including endogenous secondary metabolites, endophyte produced toxins and the hyperaccumulation of metals, all of which function as presumed defences against herbivory (Krämer 2010; Erb & Kliebenstein 2020; Whitehead *et al*. 2021). These chemical defences are thought to play a central role in antagonistic co‐evolutionary interactions between plants and insect herbivores, contributing to the diversification of both groups (Ehrlich & Raven 1964; Maron *et al*. 2019). The legume family (Fabaceae) exemplifies this pattern, as it is one of the most species‐rich plant families and is well known for both its diverse chemical defences and specialized insect herbivores (Janzen 1971). However, plants do not solely interact with herbivores, and chemical defences can potentially impact plant biotic interactions with a broad range of species including mutualists (Valdez Barillas *et al*. 2012; Schiavon & Pilon‐Smits 2017a). While antagonistic interactions between plants and herbivores have received considerable attention, less is known about the impact of chemical defences on off‐target species, particularly host‐associated microbial communities.

The hyperaccumulation of metals, including arsenic, cadmium, chromium, cobalt, copper, lead, manganese, nickel, selenium and zinc, is widespread throughout the plant kingdom, having been documented in over 750 species from 82 different plant families (Krämer 2010; Manara *et al*. 2020) and is prevalent throughout the Fabaceae (Pandey *et al*. 2022). Despite its broad occurrence throughout plant evolution, metal hyperaccumulation has received far less investigation than endogenous plant toxins (Putra & Müller 2023). This discrepancy is problematic given the dramatic increase in sites with anthropogenic metal pollution (Xiao *et al*. 2023). Hyperaccumulators are thought to possess mechanisms to actively concentrate heavy metals within plant tissues where concentrations reach 50–100x relative to surrounding non‐accumulating plant species (Verbruggen *et al*. 2009; Maestri *et al*. 2010). Metal hyperaccumulators typically inhabit soils with elevated concentrations of a particular metal, including both naturally occurring and contaminated sites such as serpentine soils and mine tailings, respectively (Cappa & Pilon‐Smits 2014; Schiavon & Pilon‐Smits 2017b). For this reason, metal hyperaccumulating plants provide a novel opportunity for bioremediation of anthropogenically contaminated soils. The vast majority of studies examining hyperaccumulation in plants have focused on nickel hyperaccumulation or more model systems such as *Arabidopsis halleri* (Pollard *et al*. 2014; Corso *et al*. 2021), with much less known about hyperaccumulation of other metals such as selenium. While selenium is an essential nutrient, it can also be a toxin (Schiavon & Pilon‐Smits 2017b). Selenium hyperaccumulation evolved independently numerous times and has been identified in 45 species across six plant families, all of which occur on naturally occurring and contaminated seleniferous soils in the western USA (Galeas *et al*. 2007). The majority of selenium hyperaccumulators (25 spp.) are in the legume genus *Astragalus* (Fabaceae), commonly referred to as milkvetches or locoweed (Schiavon & Pilon‐Smits 2017a).

Overall, there is considerable evidence suggesting a functional role for metal hyperaccumulation in herbivore defence, including both deterrence and toxicity, in support of the elemental defence hypothesis (Cappa & Pilon‐Smits 2014; Manara *et al*. 2020; Putra & Müller 2023). This is certainly the case for selenium as numerous studies, predominately in legumes, have documented that selenium hyperaccumulation provides protection against a suite of diverse herbivores (Hanson *et al*. 2003, 2004; Freeman *et al*. 2007; Quinn *et al*. 2008, 2010; El Mehdawi & Pilon‐Smits 2012; Schiavon & Pilon‐Smits 2017a, 2017b). However, there are specialist herbivores that have evolved resistance to selenium and can readily feed on hyperaccumulators (Freeman *et al*. 2012; Valdez Barillas *et al*. 2012), as is predicted by coevolutionary arms races (Maron *et al*. 2019). Other biotic interactors show a similar pattern to herbivores with some taxa being unaffected by selenium, perhaps due to evolving resistance, and others being susceptible (El Mehdawi & Pilon‐Smits 2012). For example, selenium hyperaccumulation can potentially have allelopathic effects on plant competitors (El Mehdawi *et al*. 2011), whereas pollinators are not deterred, and seemingly unimpacted, despite flowers having some of the highest levels of selenium within hyperaccumulating plants (Quinn *et al*. 2011).

Plants associate with an incredible diversity of soil microorganisms, both inside and outside of plant tissues, termed the microbiome. The microbiome has been documented to mediate nearly every conceivable plant interaction with the environment including abiotic stress tolerance (Cordovez *et al*. 2019; Gutierrez & Grillo 2022), thus plant microbiome interactions may play an important role for selenium hyperaccumulators. Selenium is found at high levels in all plant tissues of hyperaccumulators including roots (Valdez Barillas *et al*. 2012), which may disrupt key belowground plant–microbe interactions including the legume‐rhizobium mutualism (Lindblom *et al*. 2013; Etesami 2018). However, it remains unclear from the available literature what impact selenium hyperaccumulation has on the microbiome. One may predict that selenium hyperaccumulators have reduced microbiome diversity and may be enriched with selenium resistant microbes in comparison to non‐accumulating species—a similar pattern of reduced microbial diversity is observed on serpentine soils (Mengoni *et al*. 2010). Multiple culture‐based studies have identified numerous rhizosphere and endophytic fungal taxa that associate with both selenium hyperaccumulating and non‐accumulating plants and demonstrate tolerance to selenium in culture (Wangeline *et al*. 2011; Valdez Barillas *et al*. 2012; Lindblom *et al*. 2013). Less is known about the impact of selenium hyperaccumulation on bacterial microbiomes and culture‐based approaches are less adept at capturing members of the bacterial microbiome in comparison to fungal endophytes. Sura‐de Jong *et al*. (2015) found that endophytic bacterial communities from hyperaccumulating species were as diverse as non‐accumulators growing at the same seleniferous site, but this study utilized T‐RFLPs to profile the community and thus was unable to determine the identity of microbiome taxa. Using 16S rRNA amplicon sequencing, Cochran *et al*. (2018) found that the rhizosphere microbiomes of hyperaccumulators were more similar to each other than to those of non‐hyperaccumulators at the same seleniferous site. However, they did not examine endophytic microbiomes inside plant tissues where selenium concentrations are quite elevated above levels in the soil. Endophytes may be especially important for plant responses to selenium (Valdez Barillas *et al*. 2012; Lindblom *et al*. 2013). These previous microbiome studies focused primarily on hyperaccumulators inhabiting seleniferous soils. In such habitats microorganisms have likely evolved mechanisms to tolerate selenium. Yet, soils containing high metal concentration are typically quite restricted and plant species distributions can encompass a broader range including soils that are not elevated in metals (Bothe & Słomka 2017). In soils not elevated in metals, microbes are not expected to have evolved tolerance mechanisms. Thus, hyperaccumulators may have different microbiome interactions in different parts of their range. Understanding these dynamics is especially important for informing efforts to utilize metal hyperaccumulators for phytoremediation of contaminated sites (Segura *et al*. 2009; Sessitsch *et al*. 2013).

Here we examine the impacts of selenium hyperaccumulation on the root endophytic and rhizosphere microbiome of two *Astragalus* species with overlapping species distributions. *Astragalus crotalariae* is one of 25 *Astragalus* species known to hyperaccumulate selenium (Alford *et al*. 2012) and occurs on both seleniferous and non‐seleniferous soils; in contrast, *A. lentiginosus var. borreganus* is a selenium non‐accumulator. Using a manipulative greenhouse study, we grew both species on field soil and experimentally added selenium to examine if selenium hyperaccumulation has off‐target impacts on the bacterial microbiome. Specifically, we address three questions: (1) Does selenium addition alter microbiome diversity and composition across plant compartments? (2) Do selenium hyperaccumulators and non‐accumulators differ in their microbiome responses to selenium? And (3) are particular microbial taxa, especially rhizobial symbionts, disproportionately affected by selenium? We hypothesize that selenium hyperaccumulation will differentially structure the plant‐associated microbiome depending on host strategy, with non‐accumulators exhibiting greater sensitivity to selenium addition.

## METHODS

### Greenhouse experimental design

The seeds of *A. crotalariae* and *A. lentiginosus* var. *borreganus* (hereafter *A. lentiginosus*) were collected near Anza‐Borrego Desert State Park in Southern California in May of 2019. Soil for this experiment was collected in the field directly adjacent to *A. crotalariae* plants. This soil was determined to be deficient in selenium (<2.0 mg of selenium/kg of dry weight, hereafter mg kg^−1^ DW) through inductively coupled plasma‐atomic emission spectroscopy (ICP‐AES) (EMSL Analytical, Inc., Indianapolis, IN). Thus, the microbial source community is unlikely to have evolved tolerance to selenium.

Seeds of both species were surface sterilized using a 30% bleach solution, followed by multiple rinses with autoclaved water (Dotson 2025). After sterilization, seeds were scarified using sandpaper and then placed in a wet paper towel within a petri dish to germinate in a growth chamber for 3 days. Germinated seeds were subsequently transplanted into 1.5″ × 8.25″ cone‐tainers filled with field soil. After 1 week of growth, plants were divided into 4 experimental treatments with 7 plants per treatment. For the experimental treatments, plants were watered with 5 mL of sodium selenate (Na_2_SeO_4_) two times each week. The sodium selenate treatment levels included 100 μM, 80 μM, 20 μM and 0 μM. Plants were harvested after 10 weeks of growth. The bulk soil, rhizosphere, and root tissue of five plants for each unique species by sodium selenate treatment combination were selected for DNA extraction. Each compartment was extracted using DNEasy Powersoil DNA Isolation Kits (Qiagen, Hilden, Germany) according to the recommended protocol. At harvest, soil that easily fell away from the roots was used for bulk soil DNA extraction. Root tissue was then rinsed in ddH2O to remove loosely adhered soils. These roots were then placed into 50 ml conical tubes with 40 ml of sterilized milli‐Q water and vortexed for 2 min with physical agitation every 30 s to release the rhizosphere soil that was adhered to roots for DNA extraction. The clean roots were then removed from the conical tube using forceps sterilized in 70% ethanol and placed into a new 50 ml conical tube. Upon removal of the clean roots, the rhizosphere soil solution was centrifuged and the rhizosphere soil pellet was used for DNA extraction. Cleaned roots were washed with 40 ml of 40% bleach solution and subsequently rinsed three times with 40 ml of water before being used for root endosphere DNA extraction. Additionally, nodules of each species were pooled and processed the same way as roots for DNA extraction. Nodule DNA was used to determine the identity of the rhizobia that forms nodules with each species.

At the end of the experiment, leaf tissue of both *A. crotalariae* and *A. lentiginosus* in both experimental and control treatments was dried and pooled to form 1 g of dry weight. The leaflets were then sent to Indianapolis Lab of EMSL Analytical, Inc. to quantify the accumulation of selenium in the leaf tissue via inductively coupled plasma‐optical emission spectroscopy (ICP‐OES). Leaf tissue was used as opposed to root tissue as there was not sufficient root tissue for DNA extraction and selenium quantification.

### DNA sequencing and processing

The extracted, amplified bacterial DNA was sent to the Loyola Genomics Facility for library preparation and sequencing. Sequencing was performed using a single run of Illumina MiSeq (2 × 250 PE). The primer set 515F (5′‐GTG CCA GCM GCC GCG GTA A‐3′) and 806R (5′‐GGA CTA CHV GGG TWT CTA AT‐3′) was used to amplify the V4 region of the 16S rRNA genes found within each sample.

The forward and reverse primers were trimmed from the Illumina reads using *Cutadapt* (Martin 2011). The trimmed reads were also truncated (to 240 bp in the forward reads and 200 bp in the reverse reads) to both remove base pairs of low Phred Scores and maintain sufficient overlap of reads. Finally, the reads were additionally filtered such that samples with a sequence depth greater than or equal to 1,000 reads and samples with per‐base average Phred Scores greater than 30 were utilized for analysis, The filtered reads were then denoised into analysed sequence variants (ASVs) using the R package *DADA2* (Callahan *et al*. 2016). Briefly, *DADA2* models the error in Illumina reads to divisively partition each read until the reads in each cluster are consistently produced from its central sequence, that is, the ASV (Rosen *et al*. 2012). Taxonomies of each denoised ASV reference were then initially assigned using the Ribosomal Database Project 19 (RDP; http://rdp.cme.msu.edu/) to filter out any contaminating reads, including reads that corresponded to mitochondrial, chloroplast or other plant DNA. The taxonomic identity of each read was cross‐validated and confirmed using the NCBI 16S rRNA Database (Sayers *et al*. 2022). Reads that did not match any known sequences in the NCBI 16S rRNA Database were discarded. From the remaining reads, a maximum likelihood tree was produced using the R packages *msa* (Bodenhofer *et al*. 2015) and *phangorn* (Schliep 2011) and was optimized using the GTR nucleotide substitution model.

### Statistical analyses

Alpha diversity metrics, including observed taxa (Chao1 index), Shannon Evenness (H/log(S)) and Shannon Diversity (H) were calculated for each individual sample using the R package phyloseq. These metrics were analysed using anova to test for the effects of selenium treatment, plant species, compartment and their interactions. When anova results were significant, Tukey's honest significant difference (HSD) tests were used for *post hoc* pairwise comparisons among treatment groups. To examine beta diversity, the ASV × sample table was used to generate a pairwise dissimilarity matrix based on weighted Unifrac distances. A three‐way PerMANOVA was performed to examine the influence of selenium treatment, plant species and compartment on microbiome composition (*adonis* function in the R package *vegan*; R core team 2017). The order of the fixed factors was compartment, plant species and selenium treatment as a means of examining how much variance selenium treatment can explain after accounting for variance explained by plant species and compartment alone. Additionally, as the initial analysis revealed that microbial communities were distinct across plant compartments (*F*_2,44_ = 54.4435, *P* < 0.001), plant species (*F*_1,44_ = 9.5387, *P* < 0.001) and selenium treatments (*F*_3,44_ = 2.0668, *P* = 0.0431), a series of one‐way PerMANOVAs were performed separately for each compartment to examine if selenium treatment and plant species affected community. To correct for false discovery rate (FDR), the *P*‐values were corrected using the Benjamini‐Hochberg procedure using the R package *stats* (R Core Team, 2024). Pseudo‐*F*‐test statistics, raw *P*‐values and adjusted *P*‐values for each PerMANOVA are reported in Table S4.

Microbial communities were visualized using Nonmetric Multi‐Dimensional Scaling (NMDS) constructed from pairwise weighted UniFrac distance matrix as implemented using *phyloseq*. To detect significant shifts in the overall microbial communities, a series of PerMANOVAs and PERMDISPs were performed on the corresponding distance matrices of comparison. Additionally, a modified Principal Variance Component Analysis (PVCA, Boedigheimer *et al*. 2008; Heath *et al*. 2012) was employed to quantify the amount of bacterial community variance that was explained by each treatment and their interactions. Briefly, the PVCA was performed by employing the following methods: (1) taking the NMDS loading scores from the axes that explain ≥99% of the variance when linearly modelling the weighted Unifrac distance matrix by the loading score distance matrix of each predictor, (2) fitting each axis' loading scores as dependent variables and each treatment and their interactions as random effects to mixed models using restricted maximum likelihood (REML) to produce variance components, (3) weighting each variance component by the variance each axis explains, that is, the component's *R* ^2^ value for the linear model using the distance matrix of all loading scores and (4) scaling each variance by the total variance explained.

To determine differential abundance within compartments and plant species across selenium treatments, the R package *MaAsLin2* was used (Mallick *et al*. 2021). ASV tables corresponding to the plant species and compartment of interest were filtered such that the total sum of all reads for a given ASV was equal to or greater than 1,000 and were normalized using cumulative sum scaling (‘CSS’). Such normalization, utilizing a median‐like quantile normalization, was used to both maintain variation between samples and account for variation in sequencing depth across samples (Paulson *et al*. 2013). The normalized data were used in a generalized linear model to elucidate microbial features (ASVs) significantly associated with certain metadata features (selenium treatments). Only ASVs found in at least half of the tested samples and constituting at least 1% of the total relative abundance were used for the linear model. After all features were assigned *P*‐values, the Benjamini‐Hochberg procedure was performed to limit FDRs, and microbial features with q‐values <0.05 were considered significant.

## RESULTS

### The effects of selenate addition on selenium accumulation

**Fig. 1:**
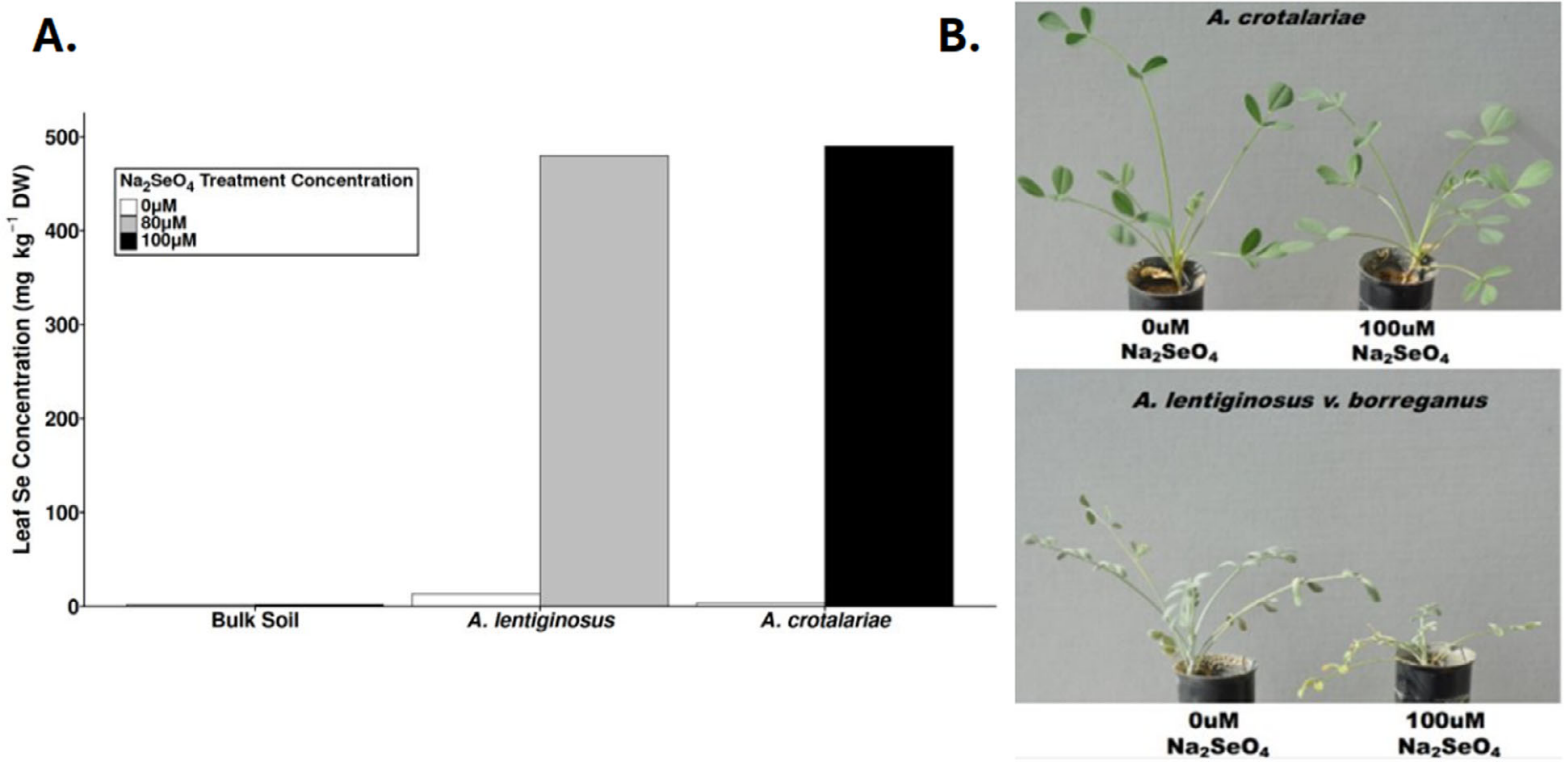
Selenium accumulation within leaf tissue. (A) Selenium concentration in the field soils prior to sodium selenate (Na_2_SeO_4_), and the leaves of the selenium hyperaccumulator *Astragalus crotalariae* and non‐hyperaccumulator *A. lentiginosus var. borreganus* after soils were treated with either no (0 μM) high (80 or 100 μM) of sodium selenate. (B) Phenotypic response of *A. crotalariae* and *A. lentiginosus* var. *Borreganus* to sodium selenate (Na_2_SeO_4_).

Field soil prior to selenate treatment and leaf tissue of plants grown on soil with and without sodium selenate were measured for selenium content to quantify the extent of selenium accumulation from the soil. In the starting bulk soil, there was little, if any, selenium present. Regardless of hyperaccumulator status, both plant species were unable to accumulate selenium from the soil when there was no added sodium selenate (Fig. 1A). The observed concentrations of selenium in both *A. lentiginosus* and *A. crotalariae* (with values <13 and 3.6 mg kg^−1^ DW, respectively) represent the reporting limit of the ICP‐AES. However, the amount of selenium added had different effects on the selenium hyperaccumulator *A*. *crotalariae* and the selenium non‐accumulator *A. lentiginosus*, whereas *A. crotalariae* seemed unaffected by the high concentration (100 μM) of sodium selenate, the treatment was lethal to *A. lentiginosus* (Fig. 1B). As such, the ‘high’ concentration for *A. crotalariae* remained at 100 μM, and the highest concentration of sodium selenate added to *A. lentiginosus* soils was reduced to 80 μM. With this change, both the selenium hyperaccumulator *A. crotalariae* and non‐accumulator *A. lentiginosus* accumulated much higher amounts of selenium in soils treated with high levels of sodium selenate than in selenium deficient soils. Most interestingly, both plants in high selenium soils had leaves with selenium concentrations (490 and 480 mg kg^−1^ for *A. lentiginosus* and *A. crotalariae*, respectively; Fig. 1A) that fall within the typical ranges of secondary selenium accumulators of 100–1000 mg of Se kg^−1^ DW in field and greenhouse studies (El Mehdawi *et al*. 2011).

### Sequencing results

Across the 59 sequenced samples, a total of 1,800,754 reads were generated, producing an average coverage of 30,522 reads per sample. After sequence filtering, processing and taxonomy assignment, a total of 4,523 ASVs were detected from 803,457 reads. A detailed sample × ASV table providing ASV abundances across all samples along with the assigned taxonomies, and representative sequences can be found in Table S1.

### The effect of selenate addition on microbial composition

Separate Principal Variance Component Analyses (PVCAs) performed for each species reveal varying responses to selenium accumulation in the hyperaccumulator and non‐hyperaccumulator microbiome. Unsurprisingly, compartment explained the largest amount of variance in bacterial community composition in both *A. crotalariae* and *A. lentiginosus*, explaining 85.98% and 80.76% of the variation in *A. crotalariae and A. lentiginosus* samples, respectively. However, the total variance explained by selenium treatment and its interaction with compartment is different, with selenium treatment and its interaction with compartment explaining 0.02% of the variation in *A. crotalariae* (Table 1A) and 6.05% of the variation in *A. lentiginosus* (Table 1B).

**Table 1:** Results of the principal variance component analysis (PVCA) for the selenium hyperaccumulator *A. crotalariae* (A) and non‐accumulator *A. lentiginosus* (B).

A				
Test (*A. crotalariae*)	Variance explained (PVCA)	Shannon diversity (H)	Shannon evenness (H/log(S))	ASV richness (S)
Selenium Concentration	0.03%	*F* = 2.699 *P* = 0.124	*F* = 0.482 *P* = 0.500	*F* = 1.642; *P* = 0.222
Compartment	96.51%	**F = 100.2;** ***P* < 0.001**	***F* = 54.841;** ***P* < 0.001**	***F* = 43.546;** ***P* < 0.001**
Selenium × Compartment	0.00%	*F* = 0.905; *P* = 0.359	*F* = 1.750; *P* = 0.209	*F* = 0.096; *P* = 0.761
Residual	3.46%			

B				
Test (*A. lentiginosus*)	Variance explained (PVCA)	Shannon diversity (H)	Shannon evenness (H/log(S))	ASV richness (S)
Selenium Concentration	**4.20%**	***F* = 16.10;** ***P* = 0.003**	***F* = 27.44;** ***P* < 0.001**	*F* = 1.544; *P* = 0.2454
Compartment	**49.71%**	***F* = 36.40;** ***P* < 0.001**	***F* = 38.24;** ***P* < 0.001**	***F* = 11.996;** ***P* = 0.007**
Selenium × Compartment	**33.37%**	***F* = 12.47;** ***P* = 0.006**	***F* = 23.79;** ***P* < 0.001**	*F* = 0.274 *P* = 0.6132
Residual	**12.72%**			

The fraction of variance explained by selenium treatment (0 μM, 20 μM, and 80/100 μM Na_2_SeO_4_; none vs. high), plant compartment (Bulk Soil, Rhizosphere and Root Endosphere) and their interaction are presented. Further, the results of the ANOVA tests of the diversity estimators are also shown. Bold squares represent significant effects on the diversity estimator corresponding to the column header.

**Fig. 2:**
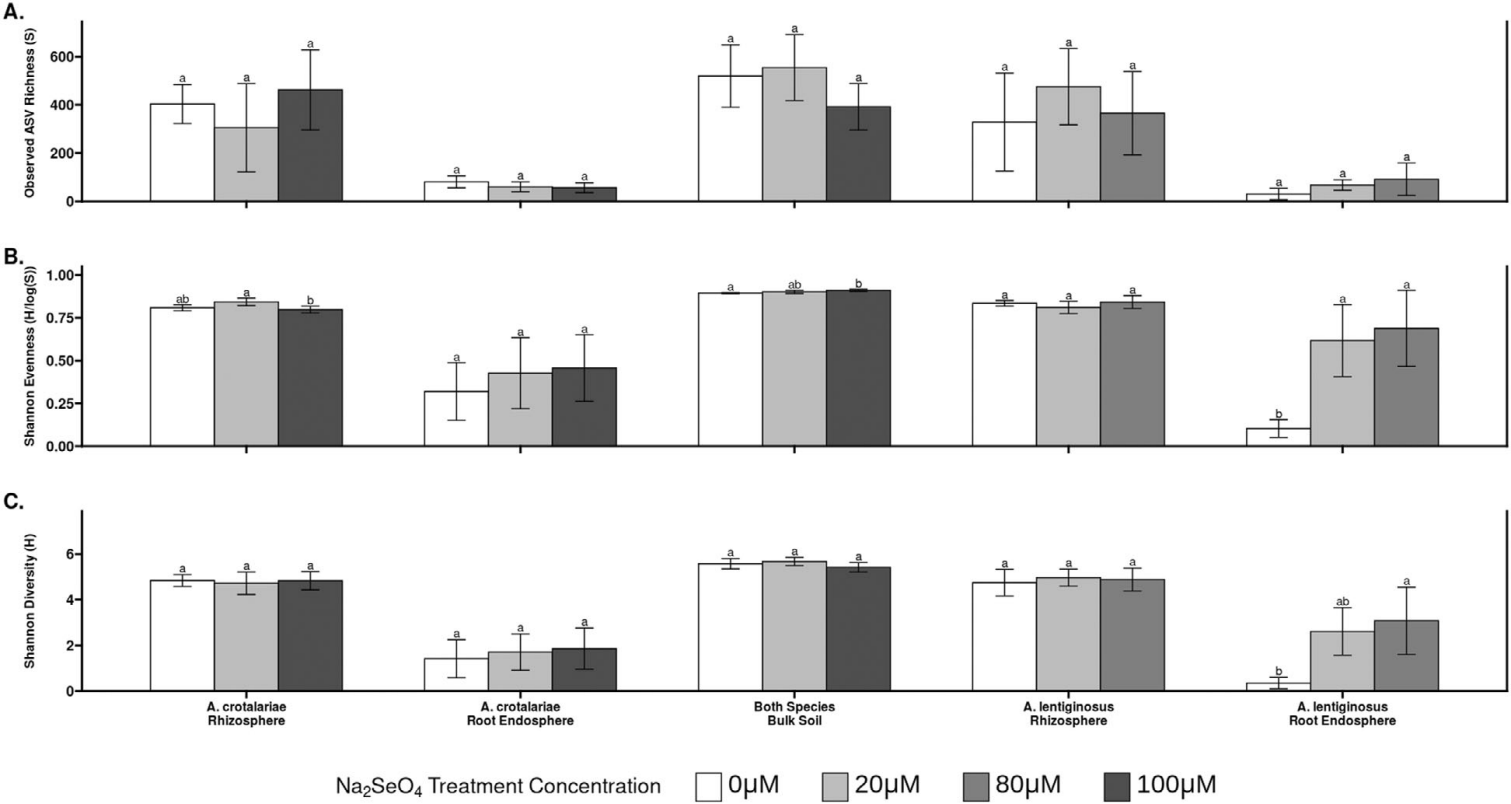
Alpha diversity metrics in response to selenium: the measured values of Chao1 diversity (richness (S)). (A) Shannon evenness (evenness (H/ln(S))); (B) and Shannon diversity (diversity (S)); (C) of the bulk soil, rhizosphere and root endosphere communities of *Astragalus crotalariae* and *A. lentiginosus* grown in soils treated with various levels of selenium.

ANOVAs on estimators of diversity, evenness and ASV richness further reveal differences in microbial response to selenium accumulation. For both *A. crotalariae* and *A. lentiginosus*, compartment significantly influenced diversity, evenness and richness of all plant‐associated communities. However, selenium concentration and its interaction with compartment differ across plant species. For *A. crotalariae*, selenium treatment was found to significantly affect community diversity (*F* = 4.739; *P* = 0.0214) and richness (*F* = 4.634; *P* = 0.023; Table 1A), whereas in *A. lentiginosus*, selenium treatment significantly affected community diversity (*F* = 4.764; *P* = 0.0250) and evenness (*F* = 8.115; *P* = 0.0041; Table 1B). Moreover, the interaction between selenium and compartment did not significantly impact community diversity, evenness or richness in *A. crotalariae*, but significantly affected diversity (*F* = 3.967; *P* = 0.0414) and evenness (*F* = 8.556; *P* = 0.0033; Table 1B) in *A. lentiginosus*. When examined within individual compartments, selenium treatment had limited effects on evenness in bulk soil communities and in the rhizosphere of *A. crotalariae*, whereas the strongest effects were observed in the root endosphere of *A. lentiginosus*, where both diversity and evenness were significantly altered (Fig. 2).

PVCA and anova using all samples produced similar results, with compartment explaining an overwhelming majority of the variance in microbial communities (compartment: 88.36%) while also having a significant effect on bacterial community diversity, evenness and ASV richness (Table S2). Moreover, the interaction of selenium treatment and plant species had a significant effect on bacterial diversity (*F* = 7.183; *P* = 0.0103; Table S2) and evenness (*F* = 7.237; *P* = 0.0101; Table S2), indicating that the bacterial communities of selenium accumulators and non‐hyperaccumulators respond differently to selenium treatment.

**Fig. 3:**
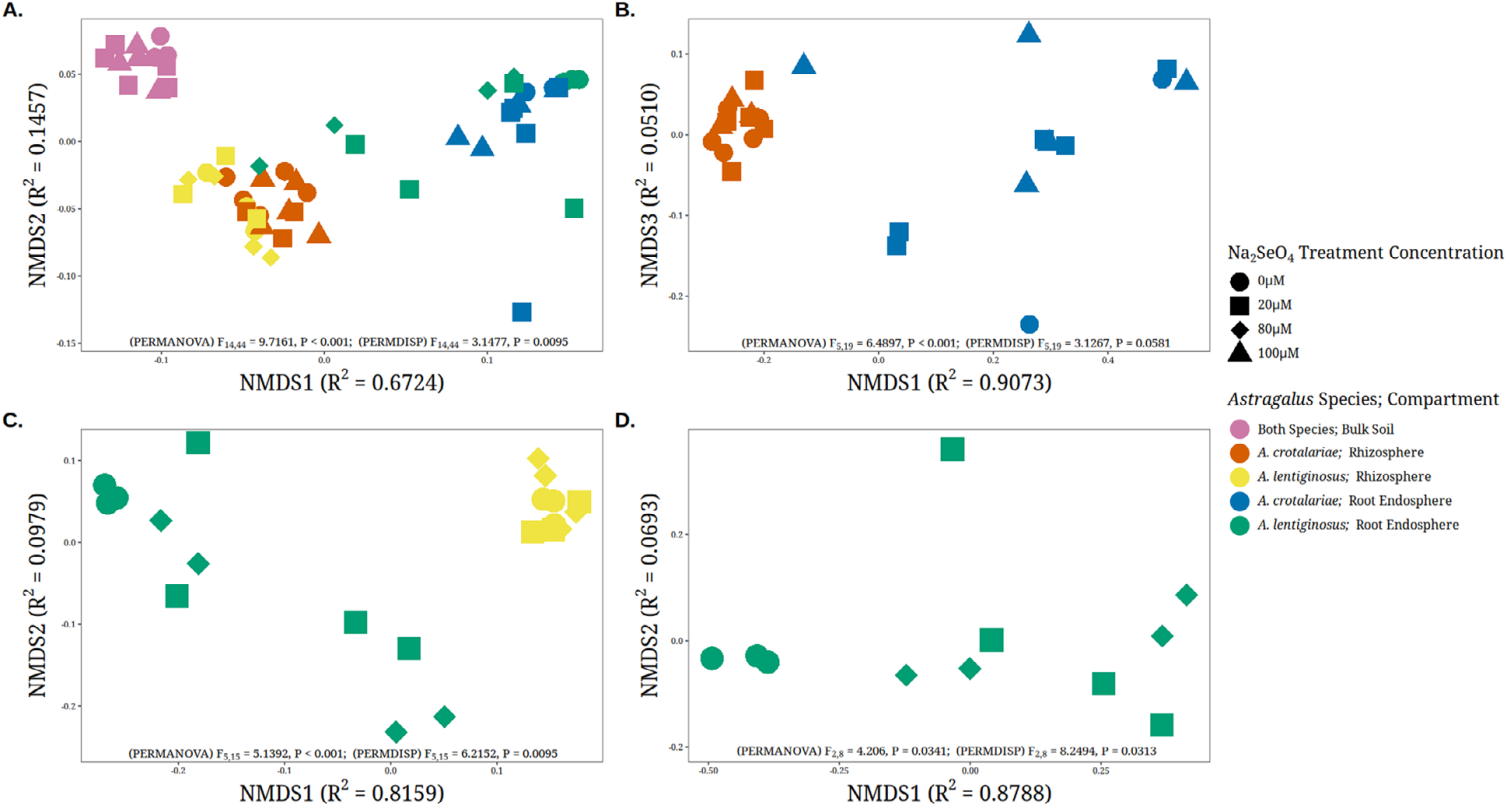
Non‐metric Multidimensional Scaling (NMDS) of all bulk soil, rhizosphere and root endosphere samples (A), samples from only *Astragalus crotalariae‐*associated communities (B) and *A. lentiginosus‐*associated communities (C), and *A. lentiginosus* root endosphere samples (D). Ordinations were produced based on weighted Unifrac distances. All *P*‐values are reported after correcting for false discovery rate using the Benjamini‐Hochberg procedure.

To delineate the effect of compartment on bacterial composition, we next employed a series of Nonmetric Multi‐Dimensional Scaling (NMDS) plots with accompanying PerMANOVAs and PermDISPs produced. Firstly, all samples were characterized by their unique compartment, treatment and associated plant species. PerMANOVA revealed at least one significant difference in bacterial community composition between all unique groups (*Pseudo F* = 9.7161, *P‐*adj < 0.001; Fig. 3A), with PermDISP detecting at least one significant difference due to differences in within‐group variance (*F =* 3.1477, *P*‐adj = 0.0095; Fig. 3A). To further interrogate the effect of selenium treatment on hyperaccumulator and non‐hyperaccumulator‐associated bacterial communities, PerMANOVA and PermDISP analyses were performed on samples associated with *A. crotalariae* and *A. lentiginosus* separately. There is at least one significant difference in bacterial communities between samples of *A. crotalariae (F* = 6.4897, *P*‐adj < 0.001; Fig. 3B) and *A. lentiginosus* (*F* = 5.1392, *P*‐adj < 0.001; Fig. 3C) when further subdivided by compartment and treatment. Additionally, all samples were further subdivided by plant species and compartment to examine the effect of selenium treatment on each individual compartment. Of the five possible plant species × compartment groupings, only the root endosphere communities of *A. lentiginosus* were found to be significantly different across selenium treatments (*F*_2,8_ = 4.206, *P*‐adj = 0.0341; Fig. 3D). However, there were no significant pairwise differences within *A. lentiginosus* root communities detected after multiple test correction (Table S3), perhaps due to significant difference in variance in *A. lentiginosus* root endosphere communities across the three treatments as detected by PermDISP (*F*_2,8_ = 8.2494; *P*‐adj = 0.0313; Fig. 3D). Nevertheless, these results strongly suggest that the root endosphere microbial communities of selenium non‐accumulators are significantly affected by selenium accumulation.

To evaluate whether observed differences between plant species were driven by plant species effects or selenium treatment, we conducted an additional analysis using only samples grown under control conditions (0 μM selenium). Within individual compartments, plant species did not exhibit significant differences in microbial community composition after correction for multiple testing (Table S5). NMDS ordination further indicated substantial overlap between species within compartments under control conditions (Fig. S1). These results suggest that baseline microbiome composition is broadly similar between species and that divergence observed under selenium treatment is likely driven by selenium addition rather than intrinsic species differences.

**Fig. 4:**
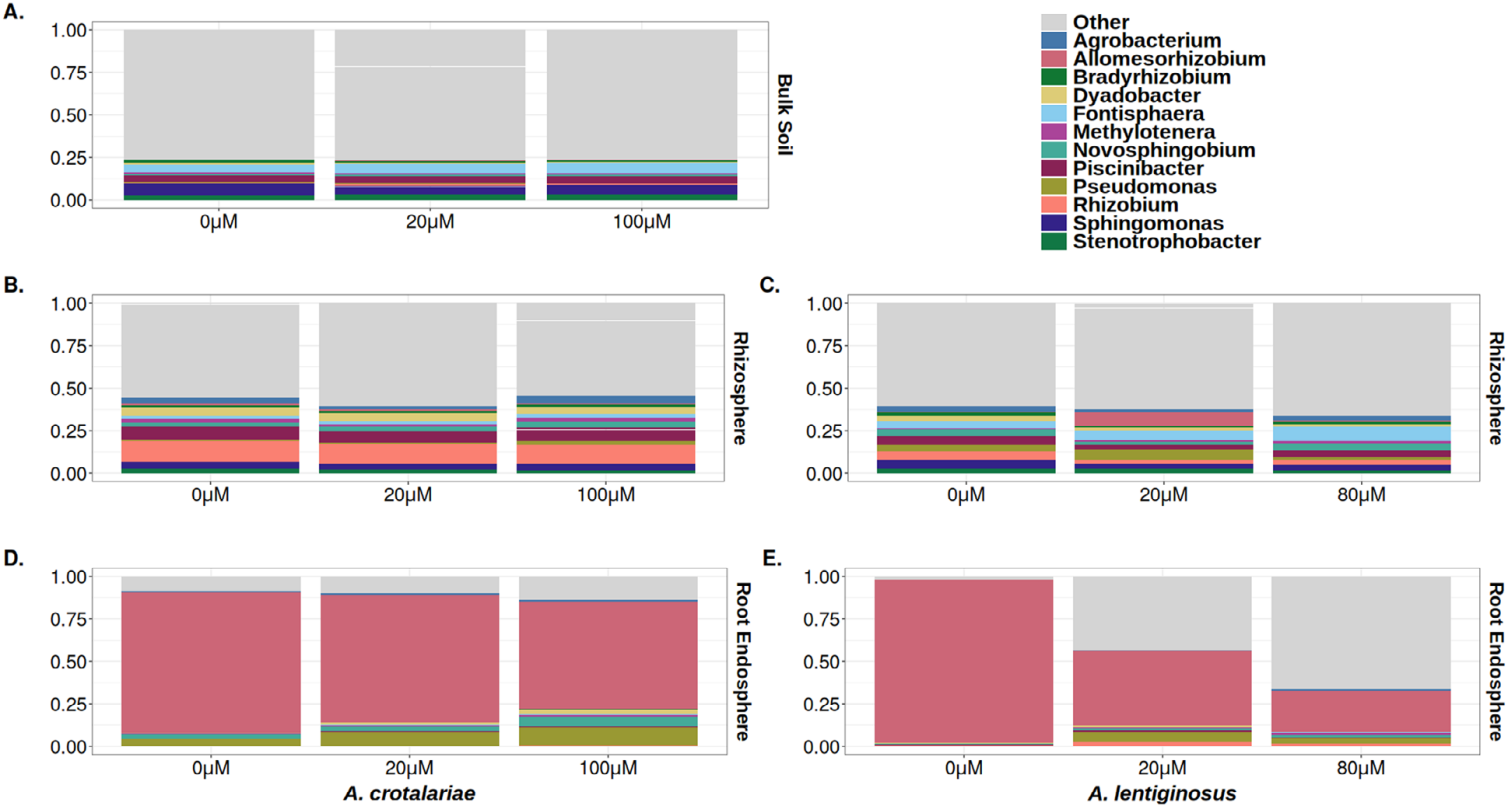
Microbial abundances of the bulk soil communities (A), the rhizosphere communities of *Astragalus crotalariae* (B) and *A. lentiginosus* (C), and the root endosphere communities of A crotalariae (D) and *A. lentiginosus* (E) in response to selenium treatments.

In terms of individual community members, ASV1 (Allomesorhizobium), corresponding to the genus that induces nodulation in *Astragalus* spp., accounted for 12.5% of all reads. Interestingly, ASV1 was either detected in low abundances or even not at all in the bulk soil (Fig. 4A) and rhizosphere communities (Fig. 4B,C), but accounted for 67.3% of all root endosphere reads (Fig. 4D,E). The remaining ASVs, spanning across 40 different phyla, were more evenly represented in the remaining reads, with relative abundances of 3.1% to less than 1%.

To examine differentially abundant ASVs within samples of the same plant species and compartment across selenium treatments, a total of five pairwise, univariate analyses for each unique plant species × compartment grouping were performed using MaAsLin2 (Mallick *et al*. 2021). The identities of the ASVs determined to be significant by MaAsLin2 are summarized in Table S4. Briefly, 84 differentially abundant ASVs were detected across all analyses. The bulk soil community held the most differentially abundant ASVs with 37 ASVs, with the majority (32) being more abundant in soils without selenium treatment (Table S4) relative to soils with any level of selenium treatment. Next, the rhizosphere communities had 29 significant differentially abundant ASVs (19 in *A. crotalariae* and 10 in *A. lentiginosus*) but were evenly distributed between selenium treatments. Finally, the root communities had 18 differentially abundant ASVs (10 in *A. crotalariae* and 8 in *A. lentiginosus*). For each plant species, there is typically a higher proportion of the differentially abundant microbes found in roots without selenium treatment than in plants grown on soils treated with selenium.

**Fig. 5:**
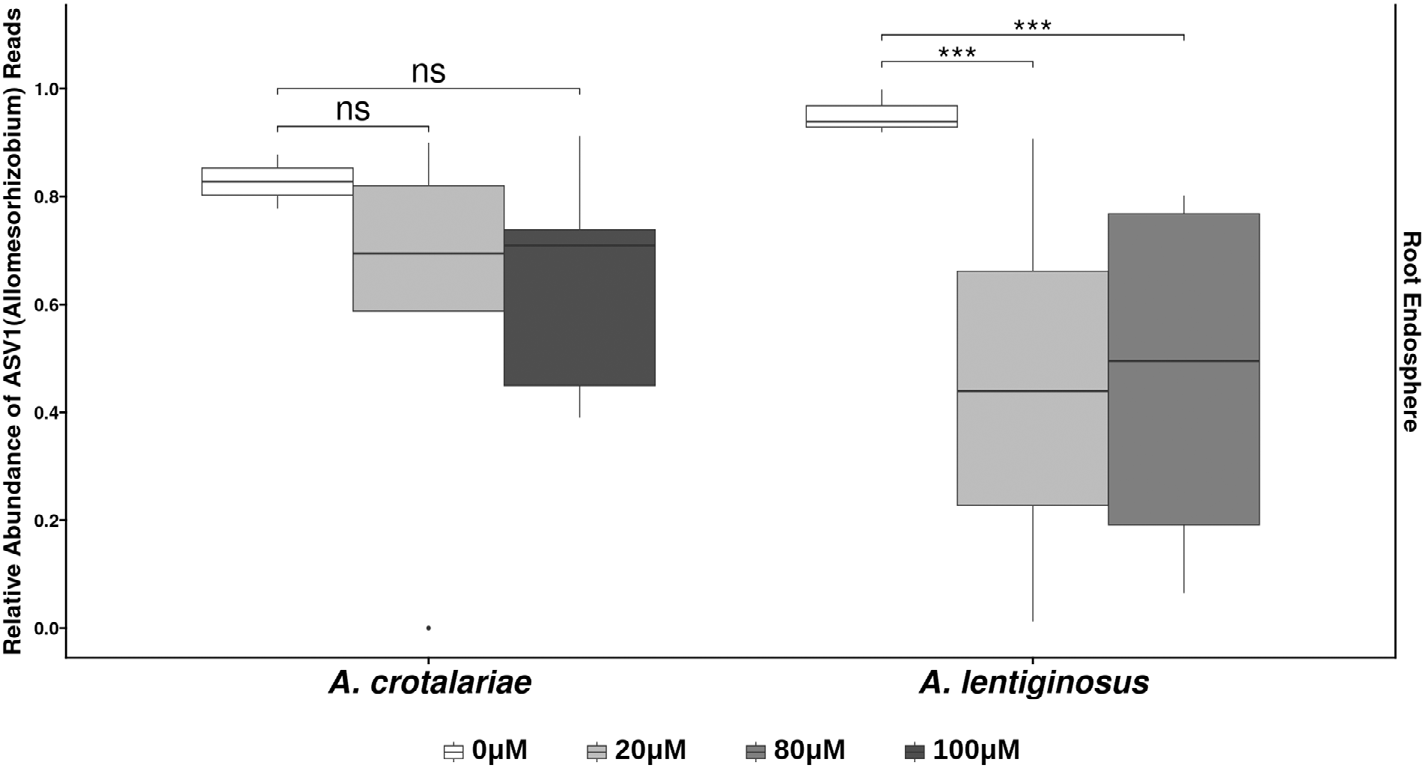
Relative differential abundance of ASV1 (*Allomesorhizobium*) in the root endosphere communities of *Astragalus crotalariae* and *A. lentiginosus*. Tests for significant differential abundance of ASV1 within plant species and compartments across selenium treatments were performed using MaAsLin2. Significance levels: ‘ns’: *P >* 0.05, ***P < 0.001.

ASV1 (*Allomesorhizobium*) exhibits an especially interesting pattern of differential abundance in both *A. crotalariae* and *A. lentiginosus*. In both species, ASV1 went from low abundance in the rhizosphere to higher abundance in the root endosphere communities (Fig. 4B,C). Within compartments across treatments, there was no significant difference in ASV1 abundance between rhizosphere communities with and without selenium treatment for both plant species (Table S4). Interestingly, ASV1 was significantly more abundant within the root endosphere communities of *A. lentiginosus* in soils without selenium relative to soils with selenium treatment (Fig. 5). Moreover, ASV34, another ASV corresponding to the genus *Allomesorhizobium*, was also found in high abundance in the nodule endosphere (Fig. S2) and displayed a similar pattern of differential expression across selenium treatments (Table S4). However, in *A. crotalariae*, ASV1 was not differentially abundant across any treatments of *A. crotalariae* root communities; rather, ASV6, identified as *Rhizobium*, which is another genus that is capable of inducing nodulation in legumes but not identified in the nodule endosphere (Fig. S2), was found to be in significantly higher abundances of *A. crotalariae* root endosphere communities with no selenium treatments. These results seem to suggest that these strains of *Allomesorhizobium* and *Rhizobium* perhaps possess little resistance to the selenium accumulated in the soil and/or in the root tissue on a per‐plant species basis.

## DISCUSSION

Metal hyperaccumulation is widespread across plant evolution, particularly within the family Fabaceae. Metal hyperaccumulation may serve as a defence mechanism against herbivory and is of significant importance for bioremediation of contaminated soils (Segura *et al*. 2009). However, metal hyperaccumulation can potentially impact non‐target biotic interactions including the microbiome. Here we examined the effect of selenium addition on the microbiomes of two co‐occurring *Astragalus* species. We find no effect of selenium addition on the overall diversity of the microbiome of the Se‐hyperaccumulator *A. crotalariae*. In contrast, selenium and its interaction with compartment significantly impacted the diversity of the microbiome for non‐accumulator *A. lentiginosus*. Moreover, selenium impacted the abundance of specific microbial taxa for both legume species, most profoundly for interactions with nitrogen‐fixing rhizobial symbionts.

A key consideration in this study is whether observed microbiome differences are driven by host species or selenium treatment. Because our experimental design includes two closely related species, we cannot fully exclude species effects. However, analyses restricted to control conditions (0 μM selenium) indicate limited evidence for strong species‐driven differences within compartments, with no significant differences in microbial community composition after correction for multiple testing (Table S5; Fig. S1). This suggests that baseline microbiome composition is broadly similar between species, and that the divergence observed under selenium addition is more consistent with a treatment‐driven response rather than intrinsic host differences.

### Selenium speciation in accumulator and non‐hyperaccumulator plants

Surprisingly, we found no difference in the total amount of selenium accumulated in the leaf tissue of our representative Se‐hyperaccumulator, *Astragalus crotalariae*, and non‐hyperaccumulator, *A. lentiginosus* (Fig. 1). However, Se‐hyperaccumulators and non‐hyperaccumulators have been documented to use similar sulphate transporters to transport inorganic selenium in the form of SeO_4_^−2^ from the roots to the plastids in leaves (Schiavon & Pilon‐Smits 2017a). Based on this information, what likely differentiates the congeners is the form in which selenium is stored. Though the employed ICP‐AES methods do not differentiate the form of selenium that is accumulated, general patterns have emerged from the literature on Se‐hyperaccumulating and non‐hyperaccumulating taxa. In hyperaccumulators, such as *Astragalus bisulcatus* and *Stanleya pinnata*, selenium is primarily stored as methylated selenocompounds including methyl‐selenocysteine (MeSeCys), γ‐glutamyl‐MeSeCys and dimethyl diselenide (Freeman *et al*. 2006); in the non‐hyperaccumulator *Stanleya elata*, selenium is primarily assimilated into non‐methylated forms including selenomethionine and selenocysteine (Lima *et al*. 2018). The higher assimilation of selenoamino acids in non‐hyperaccumulators is thought to cause selenium toxicity due to the nonspecific incorporation of selenium into proteins, resulting in denaturation and loss of function. In contrast, hyperaccumulators possess resistance to selenium as their accumulated selenocompounds are methylated and are therefore unable to be incorporated into proteins (Brown & Shrift 1982; Terry *et al*. 2000; Van Hoewyk 2013). Our findings align with this rationale as the non‐hyperaccumulator *A. lentiginosus* did not survive on soils treated with 100 μM sodium selenate, while there was no observable effect at this level on the Se‐hyperaccumulator *A. crotalariae* in this range (Fig. 1B). Therefore, while the amount of total selenium accumulated in the Se‐hyperaccumulator and non‐hyperaccumulator is similar, the selenium compounds within each congener are likely different, potentially leading to distinct interactions with the host‐associated microbiome.

### Impact of selenium on the microbiome across plant compartments

The majority of the variance in microbiome community structure in this study is explained by compartment. Plant compartment is typically identified as a major factor structuring plant‐associated microbiomes (Cordovez *et al*. 2019; Brown *et al*. 2020). Moreover, the effects of higher selenium concentrations manifest differently depending on what plant compartment a microbe exists in (Table 1A,B; Tables S2 and S3). Selenium addition did not impact the overall microbiome diversity of *A. crotalariae* across all plant compartments, with the exception of a slight effect on the evenness of the rhizosphere communities (Fig. 2B). In *A*. *lentiginosus*, however, selenium addition did increase the evenness and diversity of root endosphere communities (Fig. 2B). This result is likely due to the sharp decrease in abundance of a particularly abundant taxon or few taxa in these communities being sensitive to selenium, allowing more diverse microbes to occupy the niche of the root endosphere. Indeed, this difference in niche between these root communities can likely be attributed to the distinct forms of selenium within the plant tissues of hyperaccumulator and non‐accumulator species. The presence of non‐methylated forms of selenium within non‐accumulators may not only induce toxicity for plants, as has been previously identified (Brown & Shrift 1982; Terry *et al*. 2000; Van Hoewyk 2013), but may also restrict or promote the growth of specific members of the microbiome as well. An additional consideration is that the stronger microbiome shifts observed in *A. lentiginosus* may reflect a stress‐mediated plant response rather than a direct effect of selenium on microbial taxa. Given that *A. lentiginosus* exhibited reduced tolerance to elevated selenium relative to *A. crotalariae*, it is possible that changes in plant physiological state contributed to the restructuring of its associated microbiome.

In each plant compartment, microbes are likely to be affected by different forms of selenium compounds. Microbes found in the bulk soil are more likely to directly experience selenate, while microbes in plant‐associated communities will likely encounter a diversity of organic selenocompounds. Considering the effect of both compartment and selenium compounds could help contextualize the ecological significance of individual members in the prokaryotic communities.

In our study, we used field soil from within the native range of both species that was very low in selenium concentration (Fig. 1A), and therefore, the soil microbial communities are not preadapted to high selenate concentrations. In the bulk soil we found that selenium addition in the form of selenate, the most abundant bioavailable form in the soil (Schiavon & Pilon‐Smits 2017b), had somewhat of an impact on soil microbial community structure (Table 1; Table S3). Such a high prevalence of selenate is likely to detrimentally impact bacterial growth and development (Lindblom *et al*. 2013; Etesami 2018). Indeed, the evenness of the bulk soil community was significantly affected by soil selenium treatment (Fig. 2B), likely indicating that overabundant taxa in the soil were sensitive to soil selenate. However, these changes in evenness did not result in significant differences in bulk soil community richness (Fig. 2A), diversity (Fig. 2C) and composition (Table S3) across selenium treatments, which is in agreement with the findings of Bennett *et al*. (2024). In addition to these whole community findings, we also identified 32 ASVs that had a significantly lower abundance in soils treated with selenium compared to soils without selenium addition (Table S4). Interestingly, several of these genera have shown a contrasting pattern in other studies and were reported to have increased in abundance in selenium rich conditions. This includes *Sphingomonas* (Jiao *et al*. 2023), *Noviherbaspirillum* (Wang *et al*. 2023) and *Bradyrhizobium* (Kinkle 1994; Nemat *et al*. 2020). However, comparisons across genera in distinct studies should be approached with some caution as these methods do not resolve strain level variation which can be consequential. For example, we identified two ASVs identified as *Methylotenera* that were either in higher abundance in soils with selenium addition (ASV136) or without selenium addition (ASV130; Table S4); this example shows that even though two ASVs may be classified to the same genera, there may be some underlying strain‐specific variation that may lead to different responses to selenium addition. Additionally, there were five other genera that had significantly higher abundances in high selenate soils compared to soils with no selenium addition. These genera include *Conexibacter*, *Haliangium*, *Desulfobacca*, *Methylotenera* and *Anatilimnocola*. With the exception of *Haliangium*, which demonstrated positive growth in *Brassica chinensis* L. rhizosphere communities treated with selenium nanoparticles (SeNPs; Cheng *et al*. 2023), none of these genera have been shown to be associated with selenium/metal‐enriched soils or abiotic stress tolerance in plants. Based on our findings, these taxa are likely tolerant of inorganic selenium in the soil; however, we did not find them to be in high abundance in the rhizosphere and root communities of either species and thus are unlikely to mediate significant plant interactions with selenium.

Rhizosphere microbial communities are well recognized as being crucial for mediating plant interactions with the external soil environment and abiotic stress (Cordovez *et al*. 2019). The rhizosphere provides a complex interface of microbial activity as microbes may be faced with selenium in multiple forms. Beyond the introduced selenate molecules, the rhizosphere microbes encounter a variety of root exudates and other organic carbon sources that may contain plant‐assimilated selenium (Hartmann *et al*. 2008). Indeed, root exudates of Se‐hyperaccumulators have been documented to contain selenium, particularly in the form of organic selenocompounds (Vonderheide *et al*. 2006; El Mehdawi *et al*. 2012). Though we did not find a significant impact of selenium addition on the overall rhizosphere prokaryotic community of both *A. crotalariae* (Table S3A) and *A. lentiginosus* (Table S3B), there were individual members of the community that were significantly affected by selenium treatment. Overall, *A. lentiginosus* rhizosphere communities had a more pronounced response to selenium with a higher number of differentially abundant taxa in the rhizosphere (8 and 11 ASVs found in soils with and without Se treatment, respectively) compared to *A. crotalariae* (4 and 6 ASVs for soils with and without Se treatment, respectively; Table S4). In *A. crotalariae*, of the 10 ASVs that were differentially abundant in soils with or without selenium treatment, only the genus *Flavobacterium* has been demonstrated to be tolerant of selenium. Notably, Han *et al*. (2024) found that a strain of *Flavobacterium anhui* collected from a soil treated with 0.5 mg kg^−1^ selenate‐induced *Brassica napus* L. resistance to *Sclerotinia sclerotiorum* when in a synthetic community of various *Bacillus* and *Burkholderia* strains. Moreover, in *A. lentiginosus*, of the 19 ASVs that were differentially abundant in either soils with or without selenium treatment, only the genus *Streptomyces* has previously been associated with selenium. A variety of *Streptomyces* strains are well documented for their ability to produce SeNPs which have been demonstrated to both increase yields and protect against crop pathogens in potato (Liu *et al*. 2025). Our findings support previous findings in suggesting that these ASVs classified as *Flavobacterium* and *Streptomyces* possess some resistance to selenium. All in all, despite some overlapping genera, the members of the prokaryotic communities in the rhizospheres of each plant species responded differently to selenium treatments. While we did not measure the form of selenium in our study, it is possible that distinct forms of selenium produced by Se‐hyperaccumulator and non‐accumulator species are driving these shifts in the microbial community.

The root endosphere contains the highest concentration of selenium and, therefore, greatest potential for impacts on the microbiome. Similar to the rhizosphere, microbes within the root endosphere likely encounter distinct selenocompounds depending on the accumulator status of the host and how each host processes selenium. Interestingly, while there was no significant difference in diversity metrics for the overall root endophytic prokaryotic community across selenium treatments in *A. crotalariae* (Table S4A,B), there were pronounced differences in evenness and diversity in *A. lentiginosus*. In addition to this finding, there remain individual microbial taxa that were differentially influenced by soil selenium addition. In *A. lentiginosus*, ASVs identified as *Curvibacter*, *Novosphingobium*, *Methylotenera* and *Staphylococcus* were found in higher abundances in selenium treated soil. In a large culture‐based study, Sura‐de Jong *et al*. (2015) isolated three different *Staphylococcus* endophytes from the Se‐hyperaccumulators *Astragalus bisulcatus* and *Stanleya pinnata* that exhibited a range of tolerances to selenate and selenite. The remaining ASVs that demonstrated greater abundance with selenium addition in *A. lentiginosus* roots have not previously been reported to be associated with selenium or metal tolerance in plants.

Of the differentially abundant ASVs detected in the root endosphere communities of the selenium hyperaccumulator *A. crotalariae*, only two ASVs, both identified as members of the genus *Rhizobium*, are highly prevalent in the root endosphere communities and have been previously associated with selenium. However, these two ASVs (ASV3 and ASV6) had distinct patterns in differential abundance: where ASV6 appeared in greater abundance in the roots of *A. crotalariae* grown in soils with no selenium treatment, ASV3 was found in higher abundances in the roots growing in soils with selenium treatment (Table S4). Interestingly, *Rhizobium* has been demonstrated to have a range of tolerances to selenium (Kinkle 1994; Hunter & Kuykendall 2007). As these soils are likely to contain large, diverse populations of *Rhizobium*, it is possible that these abundance patterns between these two closely related ASVs may be the result of differing tolerances to selenium on a per‐strain basis.

### Selenium hyperaccumulation and legume–rhizobium interactions

The most pronounced impacts of selenium observed in our experiment were on *Allomesorhizobium*, the rhizobial symbiont that induces nodule formation in *Astragalus* spp. (Laguerre *et al*. 1997; Zhao *et al*. 2008). *Allomesorhizobium* was the dominant genera not only in the nodule but also in the root endosphere across all plant species and selenium treatments. A recent microbiome study in the model legume *Medicago truncatula* identified *Sinorhizobium* as the dominant microbe in the nodule, root endosphere and rhizosphere when grown on its native soil (Brown *et al*. 2020). While we found *Allomesorhizobium* to be dominant in the root endosphere (Fig. 4E) and nodule endosphere (Fig. S2), this ASV was found in relatively low abundance in the rhizosphere (Fig. 4C). Given the dominance of *Allomesorhizobium* in the root endosphere, it is possible that these symbionts mediate plant responses to selenium. Numerous studies have documented the ability of rhizobia to assist legumes in overcoming ecological stressors, including metal tolerance (reviewed in Hao *et al*. 2014). However, the functional role of rhizobia in plant tissues other than the nodule (*i.e*. the root endosphere) has not been previously considered (Brown *et al*. 2020). In many cases, the microbial mediation of metal resistance involves the microbial resistance to the stressor while also providing a plant‐growth promoting effect (Kinkle 1994). Indeed, a potential microbially mediated response to an ecological stressor such as high soil selenium levels would require microbial adaptation to the same ecological stressor (Antonovics *et al*. 1971). In the context of selenium, many rhizobial strains capable of inducing nodulation have been demonstrated to be resistant to various forms of selenium (Hunter & Kuykendall 2007; Hunter 2014; Gao *et al*. 2024). These previous findings suggest that rhizobia are likely key mutualists that can aid in resistance in selenium hyperaccumulators by resisting selenium themselves. However, in our study, the abundance of *Allomesorhizobium* was reduced in the high selenium treatment for the non‐hyperaccumulator *A. lentiginosus* (Fig. 5). This result is surprising as it has been demonstrated that not only does selenium not affect the legume‐rhizobia symbiosis in *Astragalus* hyperaccumulators and non‐accumulators in terms of nodulation rates (Alford *et al*. 2012), but also that fixed nitrogen may play a crucial role in the detoxification selenium in hyperaccumulators (Alford *et al*. 2014). These findings could be due to different forms of selenium accumulated in the root endosphere tissue; as *A. lentiginosus* likely does not possess the genetic mechanisms necessary to detoxify selenium at these ecologically relevant levels, then perhaps the accumulated selenocompounds could be lethal to the majority of the *Allomesorhizobium* strains that inhabit the root endosphere, including ASV1(*Allomesorhizobium*). In turn, the potential ability of *A. crotalariae* to detoxify selenium into methylated forms could explain why the proportion of ASV1 within the root endosphere appeared to be unaffected by selenium treatments (Fig. 5). These distinctions in rhizobial responses between selenium hyperaccumulators and non‐accumulators can be important, especially when needing to find a rhizobial symbiont in highly seleniferous soils; if the host non‐accumulator is unable to protect its symbiont from lethal doses of selenium, the symbiont cannot effectively produce the resources necessary for the host plant to compete with other plants. Thus, though it is still unclear if *Allomesorhizobium* has a role in inducing selenium tolerance in *Astragalus*, our findings suggest that the *Astragalus* host's interactions with selenium can potentially have a significant impact on their associated symbionts in seleniferous conditions.

## CONCLUSION

The native soil utilized in this experiment was very low in selenium, thus the source microbial community was likely not adapted to selenium. Therefore, our results provide a rigorous test of how selenium hyperaccumulation can impact microbiomes within the native range of these species. Overall, we find that selenium hyperaccumulation had relatively modest impacts on the microbiome and the effect was most pronounced in the non‐hyperaccumulating species, *A. lentiginosus*, presumably due to the form of plant assimilated selenium. Selenium hyperaccumulation had little impact on the overall diversity of the microbiome, but specific microbial taxa responded distinctly to selenium. Our results succeeded in identifying microbial taxa with previous implications of being responsive to selenium, while also identifying novel taxa that had not previously been studied in the context of metal hyperaccumulation or abiotic stress. Though these novel taxa are of potential interest for bioremediation of metal contaminated sites, the most pronounced impact involved the disruption of legume‐rhizobium interactions. Our results suggest that the rhizobial symbiont's performance in root endosphere communities may be susceptible to selenium concentration and are thus dependent on their host plant for protection from its toxic effects. The findings of this study are most informative for understanding how plant‐associated microbial communities respond to heavy metal contamination, which will prove invaluable in establishing best bioremediation practices.

## AUTHOR CONTRIBUTIONS

CD conducted bioinformatic and statistical analysis and wrote the manuscript. CK conducted the experiment and analysis. MG designed the experiment, conducted analysis, and wrote the manuscript.

## Supporting information


**Fig. S1.** Non‐metric Multidimensional Scaling (NMDS) of rhizosphere and root endosphere samples at 0 μM Na_2_SeO_4_ for *Astragalus crotalariae*‐associated and *A. lentiginosus‐*associated communities.

**Fig. S2.** Analysed sequence variants (ASV) abundances of the most abundant ASVs in the nodule endosphere.

**Table S2.** Results of the principal variance component analysis (PVCA) elucidating the fraction of variance explained by of selenium treatment (0, 20 and 80/100 μM Na_2_SeO_4_), plant compartment (Bulk Soil, Rhizosphere and Root Endosphere) and plant species (*Astragalus crotalariae* and *A. lentiginosus*), and all possible interactions. Further, the results of the ANOVA tests of the diversity estimators are also shown. Bold squares represent significant effects on the diversity estimator corresponding to the column header.
**Table S3**. Multiple PerMANOVA results of *Astragalus crotalariae*‐associated and *A. lentiginosus*‐associated prokaryotic communities. The first row represents the PerMANOVA result when using plant species, sample compartment, and Na_2_SeO_4_ treatment levels as predictors for the weighted Unifrac distance matrix of all samples. The remaining rows represent the results of PerMANOVA on a subset of the whole data set containing the samples of interest, that is, the result *A. crotalariae* contains all *A. crotalariae* samples across treatment and compartment, whereas 0–80 μM contains all *A. lentiginosus* root endosphere samples that received either 0 or 80 μM sodium selenate treatments.
**Table S5**. Multiple PerMANOVA results of *Astragalus crotalariae*‐associated and *A. lentiginosus*‐associated prokaryotic communities without selenium treatment. The first row represents the PerMANOVA result when plant species and compartment of samples with 0 μM of added selenate are used as predictors for the weighted Unifrac distance matrix of all samples. The remaining rows represent the results of PerMANOVA on a subset of the whole data set containing the samples of interest.

**Table S1.** Sample × analysed sequence variant (ASV) table and ASV classification table. Sheet 1 contains the assigned taxonomy of each denoised ASV and the detected microbial abundances for each sampled community. Sheet 2 contains detailed information about the source of the sequenced samples, including plant species, plant compartment and sodium selenate treatment level.

**Table S4.** Differential abundance of individual analysed sequence variants (ASVs) across selenium treatments. Each sheet contains the differentially abundant ASV specific to each unique plant species × compartment group, along with the coefficient, standard error, *P*‐value and *Q*‐value after Benjamini‐Hochberg correction of each significant differentially abundant ASV based on the generalized linear models generated by MaAsLin2 (Mallick *et al*. 2021).

## References

[plb70263-bib-0001] Alford É.R. , Lindblom S.D. , Pittarello M. , Freeman J.L. , Fakra S.C. , Marcus M.A. , Broeckling C. , Pilon‐Smits E.A.H. , Paschke M.W. (2014) Roles of rhizobial symbionts in selenium hyperaccumulation in Astragalus (Fabaceae). American Journal of Botany, 101, 1895–1905. 10.3732/ajb.1400223 25366855

[plb70263-bib-0002] Alford É.R. , Pilon‐Smits E.A.H. , Fakra S.C. , Paschke M.W. (2012) Selenium hyperaccumulation by Astragalus (Fabaceae) does not inhibit root nodule symbiosis. American Journal of Botany, 99, 1930–1941. 10.3732/ajb.1200124 23204487

[plb70263-bib-0003] Antonovics J. , Bradshaw A.D. , Turner R.G. (1971) Heavy metal tolerance in plants. In: Cragg J.B. (Ed), Advances in ecological research, Vol. 7. Academic Press, London, UK, pp 1–85. 10.1016/S0065-2504(08)60202-0

[plb70263-bib-0004] Bennett A.E. , Kelsey S. , Saup C. , Wilkins M. , Malacrinò A. (2024) Selenium alters the gene content but not the taxonomic composition of the soil microbiome. Environmental Microbiomes, 19, 92. 10.1186/s40793-024-00641-x PMC11575018 39558431

[plb70263-bib-0005] Bodenhofer U. , Bonatesta E. , Horejs‐Kainrath C. , Hochreiter S. (2015) Msa: An R package for multiple sequence alignment. Bioinformatics, 31, 3997–3999. 10.1093/bioinformatics/btv494 26315911

[plb70263-bib-0006] Boedigheimer M.J. , Wolfinger R.D. , Bass M.B. , Bushel P.R. , Chou J.W. , Cooper M. , Corton J.C. , Fostel J. , Hester S. , Lee J.S. , Liu F. , Liu J. , Qian H.‐R. , Quackenbush J. , Pettit S. , Thompson K.L. (2008) Sources of variation in baseline gene expression levels from toxicogenomics study control animals across multiple laboratories. BMC Genomics, 9, 285. 10.1186/1471-2164-9-285 PMC2453529 18549499

[plb70263-bib-0007] Bothe H. , Słomka A. (2017) Divergent biology of facultative heavy metal plants. Journal of Plant Physiology, 219, 45–61. 10.1016/j.jplph.2017.08.014 29028613

[plb70263-bib-0008] Brown S.P. , Grillo M.A. , Podowski J.C. , Heath K.D. (2020) Soil origin and plant genotype structure distinct microbiome compartments in the model legume Medicago truncatula. Microbiome, 8, 139. 10.1186/s40168-020-00915-9 PMC7523075 32988416

[plb70263-bib-0009] Brown T.A. , Shrift A. (1982) Selenium: Toxicity and tolerance in higher plants. Biological Reviews, 57, 59–84. 10.1111/j.1469-185X.1982.tb00364.x

[plb70263-bib-0010] Callahan B.J. , McMurdie P.J. , Rosen M.J. , Han A.W. , Johnson A.J.A. , Holmes S.P. (2016) DADA2: High resolution sample inference from Illumina amplicon data. Nature Methods, 13, 581–583. 10.1038/nmeth.3869 PMC4927377 27214047

[plb70263-bib-0011] Cappa J.J. , Pilon‐Smits E.A.H. (2014) Evolutionary aspects of elemental hyperaccumulation. Planta, 239, 267–275. 10.1007/s00425-013-1983-0 24463931

[plb70263-bib-0012] Cheng B. , Zhang J. , Wang C. , Li J. , Chen F. , Cao X. , Yue L. , Wang Z. (2023) Selenium nanomaterials alleviate *Brassica chinensis* L cadmium stress: Reducing accumulation, regulating microorganisms and activating glutathione metabolism. Chemosphere, 344, 140320. 10.1016/j.chemosphere.2023.140320 37775052

[plb70263-bib-0013] Cochran A.T. , Bauer J. , Metcalf J.L. , Lovecka P. , Sura de Jong M. , Warris S. , Mooijman P.J.W. , van der Meer I. , Knight R. , Pilon‐Smits E.A.H. (2018) Plant selenium Hyperaccumulation affects rhizosphere: Enhanced species richness and altered species composition. Phytobiomes Journal, 2, 82–91. 10.1094/PBIOMES-12-17-0051-R

[plb70263-bib-0014] Cordovez V. , Dini‐Andreote F. , Carrión V.J. , Raaijmakers J.M. (2019) Ecology and evolution of plant microbiomes. Annual Review of Microbiology, 73, 69–88. 10.1146/annurev-micro-090817-062524 31091418

[plb70263-bib-0015] Corso M. , An X. , Jones C.Y. , Gonzalez‐Doblas V. , Schvartzman M.S. , Malkowski E. , Willats W.G.T. , Hanikenne M. , Verbruggen N. (2021) Adaptation of Arabidopsis halleri to extreme metal pollution through limited metal accumulation involves changes in cell wall composition and metal homeostasis, 230, 669–682. 10.1111/nph.17173 33421150

[plb70263-bib-0090] Dotson, C. (2025) Exploring the ecological relevance of biotic and abiotic stressors in structuring legume microbial communities. Dissertations, 4246. https://ecommons.luc.edu/luc_diss/4246

[plb70263-bib-0016] Ehrlich P. , Raven P. (1964) Butterflies and plants: A study in coevolution. Evolution, 18, 586–608.

[plb70263-bib-0017] El Mehdawi A.F. , Cappa J.J. , Fakra S.C. , Self J. , Pilon‐Smits E.A.H. (2012) Interactions of selenium hyperaccumulators and nonaccumulators during cocultivation on seleniferous or nonseleniferous soil – The importance of having good neighbors. New Phytologist, 194, 264–277. 10.1111/j.1469-8137.2011.04043.x 22269105

[plb70263-bib-0018] El Mehdawi A.F. , Pilon‐Smits E.a.H. (2012) Ecological aspects of plant selenium hyperaccumulation. Plant Biology, 14, 1–10. 10.1111/j.1438-8677.2011.00535.x 22132825

[plb70263-bib-0019] El Mehdawi A.F. , Quinn C.F. , Pilon‐Smits E.A.H. (2011) Effects of selenium hyperaccumulation on plant–plant interactions: Evidence for elemental allelopathy? New Phytologist, 191, 120–131. 10.1111/j.1469-8137.2011.03670.x 21371042

[plb70263-bib-0020] Erb M. , Kliebenstein D.J. (2020) Plant secondary metabolites as defenses, regulators, and primary metabolites: The blurred functional Trichotomy1. Plant Physiology, 184, 39–52. 10.1104/PP.20.00433 PMC7479915 32636341

[plb70263-bib-0021] Etesami H. (2018) Bacterial mediated alleviation of heavy metal stress and decreased accumulation of metals in plant tissues: Mechanisms and future prospects. Ecotoxicology and Environmental Safety, 147, 175–191. 10.1016/j.ecoenv.2017.08.032 28843189

[plb70263-bib-0023] Freeman J.L. , Lindblom S.D. , Quinn C.F. , Fakra S. , Marcus M.A. , Pilon‐Smits E.A.H. (2007) Selenium accumulation protects plants from herbivory by Orthoptera via toxicity and deterrence. New Phytologist, 175, 490–500. 10.1111/j.1469-8137.2007.02119.x 17635224

[plb70263-bib-0024] Freeman J.L. , Marcus M.A. , Fakra S.C. , Devonshire J. , McGrath S.P. , Quinn C.F. , Pilon‐Smits E.A.H. (2012) Selenium Hyperaccumulator plants Stanleya pinnata and Astragalus bisulcatus are colonized by Se‐resistant, Se‐excluding wasp and beetle seed herbivores. PLoS One, 7, e50516. 10.1371/journal.pone.0050516 PMC3513300 23226523

[plb70263-bib-0025] Freeman J.L. , Zhang L.H. , Marcus M.A. , Fakra S. , McGrath S.P. , Pilon‐Smits E.A.H. (2006) Spatial imaging, speciation, and quantification of selenium in the Hyperaccumulator plants *Astragalus bisulcatus* and *Stanleya pinnata* . Plant Physiology, 142, 124–134. 10.1104/pp.106.081158 PMC1557614 16920881

[plb70263-bib-0027] Galeas M.L. , Zhang L.H. , Freeman J.L. , Wegner M. , Pilon‐Smits E.A.H. (2007) Seasonal fluctuations of selenium and sulfur accumulation in selenium hyperaccumulators and related nonaccumulators. New Phytologist, 173, 517–525. 10.1111/j.1469-8137.2006.01943.x 17244046

[plb70263-bib-0028] Gao H. , Ji Y. , Chen W. (2024) Selenite resistance and biotransformation to SeNPs in *Sinorhizobium meliloti* 1021 and the synthetic promotion on alfalfa growth. Microbiological Research, 280, 127568. 10.1016/j.micres.2023.127568 38118306

[plb70263-bib-0029] Gutierrez A. , Grillo M.A. (2022) Effects of domestication on plant–microbiome interactions. Plant and Cell Physiology, 63, 1654–1666. 10.1093/pcp/pcac108 35876043

[plb70263-bib-0030] Han C. , Cheng Q. , Du X. , Liang L. , Fan G. , Xie J. , Wang X. , Tang Y. , Zhang H. , Hu C. , Zhao X. (2024) Selenium in soil enhances resistance of oilseed rape to *Sclerotinia sclerotiorum* by optimizing the plant microbiome. Journal of Experimental Botany, 75, 5768–5789. 10.1093/jxb/erae238 38809805

[plb70263-bib-0031] Hanson B. , Garifullina G.F. , Lindblom S.D. , Wangeline A. , Ackley A. , Kramer K. , Norton A.P. , Lawrence C.B. , Pilon‐Smits E.A.H. (2003) Selenium accumulation protects *Brassica juncea* from invertebrate herbivory and fungal infection. New Phytologist, 159, 461–469. 10.1046/j.1469-8137.2003.00786.x 33873368

[plb70263-bib-0032] Hanson B. , Lindblom S.D. , Loeffler M.L. , Pilon‐Smits E.A.H. (2004) Selenium protects plants from phloem‐feeding aphids due to both deterrence and toxicity. New Phytologist, 162, 655–662. 10.1111/j.1469-8137.2004.01067.x 33873760

[plb70263-bib-0033] Hao X. , Taghavi S. , Xie P. , Orbach M.J. , Alwathnani H.A. , Rensing C. , Wei G. (2014) Phytoremediation of heavy and transition metals aided by legume‐rhizobia Symbiosis. International Journal of Phytoremediation, 16, 179–202. 10.1080/15226514.2013.773273 24912209

[plb70263-bib-0034] Hartmann A. , Rothballer M. , Schmid M. (2008) Lorenz Hiltner, a pioneer in rhizosphere microbial ecology and soil bacteriology research. Plant and Soil, 312, 7–14. 10.1007/s11104-007-9514-z

[plb70263-bib-0035] Heath K.D. , Burke P.V. , Stinchcombe J.R. (2012) Coevolutionary genetic variation in the legume‐rhizobium transcriptome. Molecular Ecology, 21, 4735–4747. 10.1111/j.1365-294X.2012.05629.x 22672103

[plb70263-bib-0038] Hunter W.J. (2014) A rhizobium selenitireducens protein showing selenite reductase activity. Current Microbiology, 68, 311–316. 10.1007/s00284-013-0474-7 24474405

[plb70263-bib-0039] Hunter W.J. , Kuykendall L.D. (2007) Reduction of selenite to elemental red selenium by rhizobium sp. strain B1. Current Microbiology, 55, 344–349. 10.1007/s00284-007-0202-2 17882505

[plb70263-bib-0040] Janzen D. (1971) Seed predation by animals. Annual Review of Ecology and Systematics, 2, 465–492.

[plb70263-bib-0042] Jiao L. , Cao X. , Wang C. , Chen F. , Zou H. , Yue L. , Wang Z. (2023) Crosstalk between in situ root exudates and rhizobacteria to promote rice growth by selenium nanomaterials. Science of the Total Environment, 878, 163175. 10.1016/j.scitotenv.2023.163175 37003329

[plb70263-bib-0043] Kinkle B.K. (1994) Tellurium and Selenium Resistance in Rhizobia and Its Potential Use for Direct Isolation of Rhizobium meliloti from Soil. 10.1128/aem.60.5.1674-1677.1994 PMC201536 16349263

[plb70263-bib-0045] Krämer U. (2010) Metal Hyperaccumulation in plants. Annual Review of Plant Biology, 61, 517–534. 10.1146/annurev-arplant-042809-112156 20192749

[plb70263-bib-0046] Laguerre G. , Van Berkum P. , Amarger N. , Prévost D. (1997) Genetic diversity of rhizobial symbionts isolated from legume species within the genera *Astragalus*, *Oxytropis*, and *Onobrychis* . Applied and Environmental Microbiology, 63, 4748–4758. 10.1128/aem.63.12.4748-4758.1997 PMC168797 9406393

[plb70263-bib-0048] Lima L.W. , Pilon‐Smits E.A.H. , Schiavon M. (2018) Mechanisms of selenium hyperaccumulation in plants: A survey of molecular, biochemical and ecological cues. Biochimica et Biophysica Acta (BBA) ‐ General Subjects, 1862, 2343–2353. 10.1016/j.bbagen.2018.03.028 29626605

[plb70263-bib-0049] Lindblom S.D. , Valdez‐Barillas J.R. , Fakra S.C. , Marcus M.A. , Wangeline A.L. , Pilon‐Smits E.A.H. (2013) Influence of microbial associations on selenium localization and speciation in roots of *Astragalus* and *Stanleya* hyperaccumulators. Environmental and Experimental Botany, 88, 33–42. 10.1016/j.envexpbot.2011.12.011

[plb70263-bib-0050] Liu H. , Zhang Y. , Zhang L. , Liu Y. , Chen Y. , Shi Y. (2025) Nano‐selenium strengthens potato resistance to potato scab induced by *Streptomyces* spp., increases yield, and elevates tuber quality by influencing rhizosphere microbiomes. Frontiers in Plant Science, 16, 1523174. 10.3389/fpls.2025.1523174 PMC11830815 39963528

[plb70263-bib-0053] Maestri E. , Marmiroli M. , Visioli G. , Marmiroli N. (2010) Metal tolerance and hyperaccumulation: Costs and trade‐offs between traits and environment. Environmental and Experimental Botany, 68, 1–13. 10.1016/j.envexpbot.2009.10.011

[plb70263-bib-0054] Mallick H. , Rahnavard A. , McIver L.J. , Ma S. , Zhang Y. , Nguyen L.H. , Tickle T.L. , Weingart G. , Ren B. , Schwager E.H. , Chatterjee S. , Thompson K.N. , Wilkinson J.E. , Subramanian A. , Lu Y. , Waldron L. , Paulson J.N. , Franzosa E.A. , Bravo H.C. , Huttenhower C. (2021) Multivariable association discovery in population‐scale meta‐omics studies. PLoS Computational Biology, 17, e1009442. 10.1371/journal.pcbi.1009442 PMC8714082 34784344

[plb70263-bib-0055] Manara A. , Fasani E. , Furini A. , DalCorso G. (2020) Evolution of the metal hyperaccumulation and hypertolerance traits. Plant, Cell & Environment, 43, 2969–2986. 10.1111/pce.13821 32520430

[plb70263-bib-0056] Maron J.L. , Agrawal A.A. , Schemske D.W. (2019) Plant–herbivore coevolution and plant speciation. Ecology, 100, 1–11. 10.1002/ecy.2704 30916391

[plb70263-bib-0057] Martin M. (2011) Cutadapt removes adapter sequences from high‐throughput sequencing reads. EMBnet.Journal, 17, 1. 10.14806/ej.17.1.200

[plb70263-bib-0059] Mengoni A. , Schat H. , Vangronsveld J. (2010) Plants as extreme environments? Ni‐resistant bacteria and Ni‐hyperaccumulators of serpentine flora. Plant and Soil, 331, 5–16. 10.1007/s11104-009-0242-4

[plb70263-bib-0060] Nemat H. , Shah A.A. , Akram W. , Ramzan M. , Yasin N.A. (2020) Ameliorative effect of co‐application of *Bradyrhizobium japonicum* EI09 and Se to mitigate chromium stress in *Capsicum annum* L. International Journal of Phytoremediation, 22, 1396–1407. 10.1080/15226514.2020.1780412 32608249

[plb70263-bib-0061] Pandey A.K. , Zorić L. , Sun T. , Karanović D. , Fang P. , Borišev M. , Wu X. , Luković J. , Xu P. (2022) The anatomical basis of heavy metal responses in legumes and their impact on plant‐rhizosphere interactions. Plants, 11, 2554. 10.3390/plants11192554 PMC9572132 36235420

[plb70263-bib-0062] Paulson J.N. , Stine O.C. , Bravo H.C. , Pop M. (2013) Robust methods for differential abundance analysis in marker gene surveys. Nature Methods, 10, 1200–1202. 10.1038/nmeth.2658m PMC4010126 24076764

[plb70263-bib-0063] Pollard A.J. , Reeves R.D. , Baker A.J.M. (2014) Facultative hyperaccumulation of heavy metals and metalloids. Plant Science, 217–218, 8–17. 10.1016/j.plantsci.2013.11.011 24467891

[plb70263-bib-0065] Putra R. , Müller C. (2023) Extending the elemental defence hypothesis in the light of plant chemodiversity. New Phytologist, 239, 1545–1555. 10.1111/nph.19071 37309036

[plb70263-bib-0066] Quinn C.F. , Freeman J.L. , Galeas M.L. , Klamper E.M. , Pilon‐Smits E.A.H. (2008) The role of selenium in protecting plants against prairie dog herbivory: Implications for the evolution of selenium hyperaccumulation. Oecologia, 155, 267–275. 10.1007/s00442-007-0907-8 18278517

[plb70263-bib-0067] Quinn C.F. , Freeman J.L. , Reynolds R.J. , Cappa J.J. , Fakra S.C. , Marcus M.A. , Lindblom S.D. , Quinn E.K. , Bennett L.E. , Pilon‐Smits E.A. (2010) Selenium hyperaccumulation offers protection from cell disruptor herbivores. BMC Ecology, 10, 19. 10.1186/1472-6785-10-19 PMC2940900 20799959

[plb70263-bib-0068] Quinn C.F. , Prins C.N. , Freeman J.L. , Gross A.M. , Hantzis L.J. , Reynolds R.J.B. , Yang S. , Covey P.A. , Bañuelos G.S. , Pickering I.J. , Fakra S.C. , Marcus M.A. , Arathi H.S. , Pilon‐Smits E.A.H. (2011) Selenium accumulation in flowers and its effects on pollination. New Phytologist, 192, 727–737. 10.1111/j.1469-8137.2011.03832.x 21793829

[plb70263-bib-0069] Rosen M.J. , Callahan B.J. , Fisher D.S. , Holmes S.P. (2012) Denoising PCR‐amplified metagenome data. BMC Bioinformatics, 13, 283. 10.1186/1471-2105-13-283 PMC3563472 23113967

[plb70263-bib-0070] Sayers E.W. , Bolton E.E. , Brister J.R. , Canese K. , Chan J. , Comeau D.C. , Connor R. , Funk K. , Kelly C. , Kim S. , Madej T. , Marchler‐Bauer A. , Lanczycki C. , Lathrop S. , Lu Z. , Thibaud‐Nissen F. , Murphy T. , Phan L. , Skripchenko Y. , Tse T. , Wang J. , Williams R. , Trawick B.W. , Pruitt K.D. , Sherry S.T. (2022) Database resources of the national center for biotechnology information. Nucleic Acids Research, 50(D1), D20–D26. 10.1093/nar/gkab1112 PMC8728269 34850941

[plb70263-bib-0071] Schiavon M. , Pilon‐Smits E.A.H. (2017a) Selenium biofortification and phytoremediation Phytotechnologies: A review. Journal of Environmental Quality, 46, 10–19. 10.2134/jeq2016.09.0342 28177413

[plb70263-bib-0072] Schiavon M. , Pilon‐Smits E.A.H. (2017b) The fascinating facets of plant selenium accumulation – Biochemistry, physiology, evolution and ecology. New Phytologist, 213, 1582–1596. 10.1111/nph.14378 27991670

[plb70263-bib-0073] Schliep K. (2011) Phangorn: Phylogenetic analysis in R. Bioinformatics, 27, 592–593. 10.1093/bioinformatics/btq706 PMC3035803 21169378

[plb70263-bib-0074] Segura A. , Rodríguez‐Conde S. , Ramos C. , Ramos J.L. (2009) Bacterial responses and interactions with plants during rhizoremediation. Microbial Biotechnology, 2, 452–464. 10.1111/j.1751-7915.2009.00113.x PMC3815906 21255277

[plb70263-bib-0075] Sessitsch A. , Kuffner M. , Kidd P. , Vangronsveld J. , Wenzel W.W. , Fallmann K. , Puschenreiter M. (2013) The role of plant‐associated bacteria in the mobilization and phytoextraction of trace elements in contaminated soils. Soil Biology and Biochemistry, 60, 182–194. 10.1016/j.soilbio.2013.01.012 PMC3618436 23645938

[plb70263-bib-0076] Sura‐de Jong M. , Reynolds R.J.B. , Richterova K. , Musilova L. , Staicu L.C. , Chocholata I. , Cappa J.J. , Taghavi S. , van der Lelie D. , Frantik T. , Dolinova I. , Strejcek M. , Cochran A.T. , Lovecka P. , Pilon‐Smits E.A.H. (2015) Selenium hyperaccumulators harbor a diverse endophytic bacterial community characterized by high selenium resistance and plant growth promoting properties. Frontiers in Plant Science, 6, 113. 10.3389/fpls.2015.00113 PMC4345804 25784919

[plb70263-bib-0077] Terry N. , Zayed A.M. , de Souza M.P. , Tarun A.S. (2000) Selenium in higher plants. Annual Review of Plant Biology, 51, 401–432. 10.1146/annurev.arplant.51.1.401 15012198

[plb70263-bib-0078] Valdez Barillas J.R. , Quinn C.F. , Freeman J.L. , Lindblom S.D. , Fakra S.C. , Marcus M.A. , Gilligan T.M. , Alford É.R. , Wangeline A.L. , Pilon‐Smits E.A.H. (2012) Selenium distribution and speciation in the Hyperaccumulator Astragalus bisulcatus and associated ecological partners. Plant Physiology, 159, 1834–1844. 10.1104/pp.112.199307 PMC3425216 22645068

[plb70263-bib-0079] Van Hoewyk D. (2013) A tale of two toxicities: Malformed selenoproteins and oxidative stress both contribute to selenium stress in plants. Annals of Botany, 112, 965–972. 10.1093/aob/mct163 PMC3783228 23904445

[plb70263-bib-0080] Verbruggen N. , Hermans C. , Schat H. (2009) Molecular mechanisms of metal hyperaccumulation in plants. New Phytologist, 181, 759–776. 10.1111/j.1469-8137.2008.02748.x 19192189

[plb70263-bib-0081] Vonderheide A.P. , Mounicou S. , Meija J. , Henry H.F. , Caruso J.A. , Shann J.R. (2006) Investigation of selenium‐containing root exudates of Brassica juncea using HPLC‐ICP‐MS and ESI‐qTOF‐MS. Analyst, 131, 33–40. 10.1039/B510712A 16365660

[plb70263-bib-0083] Wang Y. , Xu C. , Wuriyanghan H. , Lei Z. , Tang Y. , Zhang H. , Zhao X. (2023) Exogenous selenium endows salt‐tolerant and salt‐sensitive soybeans with salt tolerance through plant‐microbial coactions. Agronomy, 13, 9. 10.3390/agronomy13092271

[plb70263-bib-0084] Wangeline A.L. , Valdez J.R. , Lindblom S.D. , Bowling K.L. , Reeves F.B. , Pilon‐Smits E.A.H. (2011) Characterization of rhizosphere fungi from selenium hyperaccumulator and nonhyperaccumulator plants along the eastern Rocky Mountain front range. American Journal of Botany, 98, 1139–1147. 10.3732/ajb.1000369 21730338

[plb70263-bib-0085] Whitehead S.R. , Bass E. , Corrigan A. , Kessler A. , Poveda K. (2021) Interaction diversity explains the maintenance of phytochemical diversity. Ecology Letters, 24, 1205–1214. 10.1111/ele.13736 33783114

[plb70263-bib-0087] Xiao C.Q. , Zheng C.L. , Zhang Y.F. , He H. , Ilyas S. (2023) Editorial: Application of microbial technology in ecological remediation of mines. Frontiers in Microbiology, 14, 1136851. 10.3389/fmicb.2023.1136851 PMC9969150 36860484

[plb70263-bib-0088] Zhao C.T. , Wang E.T. , Chen W.F. , Chen W.X. (2008) Diverse genomic species and evidences of symbiotic gene lateral transfer detected among the rhizobia associated with Astragalus species grown in the temperate regions of China. FEMS Microbiology Letters, 286, 263–273. 10.1111/j.1574-6968.2008.01282.x 18657113

