# Supplementary material for: Effect of selenium hyperaccumulation on the root endophytic and rhizosphere microbiome of two Astragalus species
Source: Plant Biol (Stuttg). 2026 Jul 13;28(6):1960–72. doi: 10.1111/plb.70263 (PMC13586643; doi:10.1111/plb.70263)
Supplement: Supplementary file 1 [file PLB-28-1960-s004.pdf]

Supplemental Information

Table S1: Sample x ASV Table and ASV Classification Table. Sheet 1 contains the assigned taxonomy of each detected ASV and the detected microbial abundances for each sampled community. Sheet 2 contains detailed information about the source of the sequenced samples, including plant species, plant compartment, and sodium selenate treatment level. SEE ATTACHED DOCUMENT.

**Test**	R^2	**Variance Explained (PVCA)**	**Shannon Diversity (H)**	**Shannon Evenness (H/log(S))**	**ASV Richness (S)**
Selenium Concentration	0.034	1.86%	F = 2.801; P = 0.0509	**F =4.120; P = 0.0117**	F = 0.351; P = 0.7883
Compartment	0.645	88.36%	**F = 156.042; P < 0.001**	**F= 75.120; P < 0.001**	**F = 65.922; P < 0.001**
Species	0.046	0.53%	**F = 12.190; P = 0.0011**	F = 3.436; P = 0.0705	**F = 10.885; P = 0.0019**
Selenium x Compartment	0.037	0.03%	**F = 2.441; P = 0.0489**	**F = 3.669 P = 0.0073**	F = 1.745; P = 0.1443
Selenium x Species	0.008	1.63%	**F = 7.183; P = 0.0103**	**F = 7.237; P = 0.0101**	F = 1.506; P = 0.2263
Compartment x Species	0.012	0.43%	F = 0.824; P = 0.3689	F = 0.809; P = 0.3733	F = 0.036; P = 0.85
Selenium x Compartment x Species	0.006	0.16%	F = 2.371; P = 0.1308	**F = 5.884; P = 0.0195**	F = 1.050; P = 0.3110
Residual	0.244	7.01%			

Table S2: Results of the Principal Variance Component Analysis (PVCA) elucidating the fraction of variance explained by of selenium treatment (0, 20, 80, and 100 µM $NaSe_4O$), plant compartment (Bulk Soil, Rhizosphere, and Root Endosphere), and plant species (*A. crotalariae* and *A. lentiginosus*), and all possible interactions. Further, the results of the ANOVA tests of the diversity estimators are also shown. Bold squares represent significant effects on the diversity estimator corresponding to the column header.

Test	Pseudo-F Statistic	P-Value	Adjusted P-Value
**All Samples**	**$F_{14,44} = 9.7161$**	**P < 0.001**	**P-adj < 0.001**
Bulk Soil	$F_{2,10} = 1.2791$	P = 0.1399	P-adj = 0.1829
A. crotalariae	**$F_{5,19} = 6.4897$**	**P < 0.001**	**P-adj < 0.001**
Rhizosphere	$F_{2,10} = 1.0357$	P = 0.4025	P-adj = 0.4562
Root Endosphere	$F_{2,9} = 0.5791$	P = 0.829	P-adj = 0.8362
A. lentiginosus	**$F_{5,15} = 5.1392$**	**P < 0.001**	**P-adj < 0.001**
Rhizosphere	$F_{2,7} = 1.2605$	P = 0.2506	P-adj = 0.3042
**Root Endosphere**	**$F_{2,8} = 4.2096$**	**P = 0.0119**	**P-adj = 0.0289**
0-80µM	$F_{1,5} = 8.0003$	P = 0.0571	P-adj = 0.0901
0-20µM	$F_{1,5} = 7.3321$	P = 0.0286	P-adj = 0.0607
20-80µM	$F_{1,6} = 0.5529$	P = 0.8362	P-adj = 0.8362

Table S3: Multiple PerMANOVA Results of *A. crotalariae* and *A. lentiginosus* Associated Prokaryotic Communities. The first row represents the PerMANOVA result when using plant species, sample compartment, and sodium selenate treatment levels as predictors for the weighted Unifrac distance matrix of all samples. The remaining rows represent the results of PerMANOVA on a subset of the whole data set containing the samples of interest, i.e. the result *A. crotalariae* contains all *A.crotalariae* samples across treatment and compartment, while 0-80µM contains all *A. lentiginosus* root endosphere samples that received either 0 or 80µM sodium selenate treatments.

Table S4: Differential Abundance of Individual ASVs Across Selenium Treatments. Each sheet contains the differentially abundant ASV specific to each unique plant species X compartment group, along with the coefficient, standard error, P-value, and Q-value after Benjamini-Hochberg correction of each significant differentially abundant ASV based on the generalized linear models generated by MaAsLin2 (Mallick et al., 2021). SEE SEPARATE FILE

**Test**	**Pseudo-F Statistic**	**P-Value**	**Adjusted P-Value**
**All 0 µM Samples**	**$F_{3,12} = 23.916$**	**$P < 0.001$**	**$P < 0.001$**
Rhizosphere	$F_{1,7} = 2.4733$	$P = 0.035$	$P = 0.0661$
Root Endosphere	$F_{1,4} = 3.2513$	$P = 0.1$	$P = 0.1417$

Table S5: Multiple PerMANOVA Results of *A. crotalariae* and *A. lentiginosus* Associated Prokaryotic Communities Without Selenium Treatment. The first row represents the PerMANOVA result when plant species and compartment of samples with 0 µM of added selenate are used as predictors for the weighted Unifrac distance matrix of all samples. The remaining rows represent the results of PerMANOVA on a subset of the whole data set containing the samples of interest.
